# Supplementary figures and images for: A Protocol for the Acquisition of Comprehensive Proteomics Data from Single Cases Using Formalin-Fixed Paraffin Embedded Sections
Source: Methods Protoc. 2022 Jul 10;5(4):57. doi: 10.3390/mps5040057 (PMC9326557; doi:10.3390/mps5040057)

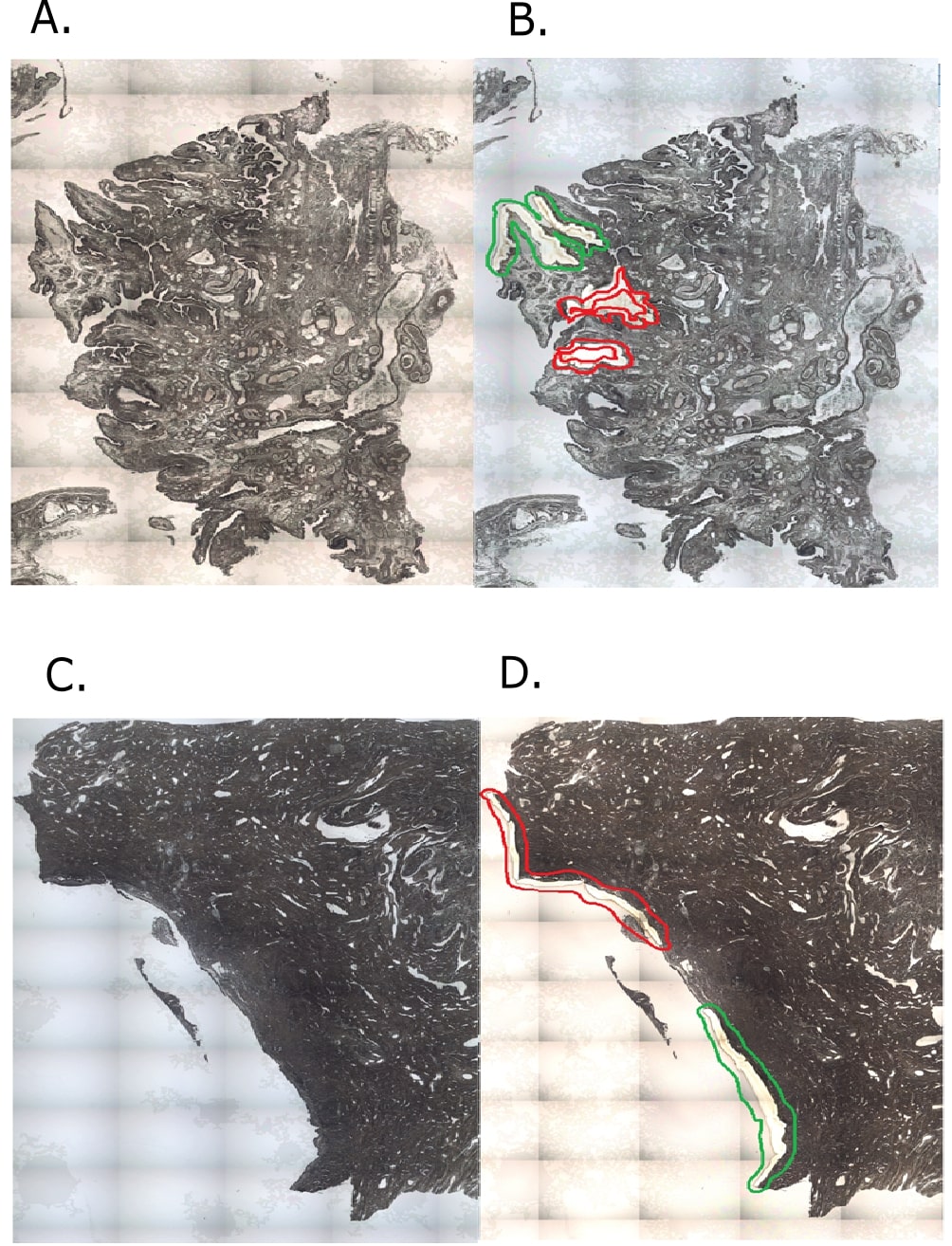

Supplement: Supplementary file 1 [file mps-05-00057-s001.zip › Supplementary Figure 1.jpg]
